# Motion Artifact Reduction Using U-Net Model with Three-Dimensional Simulation-Based Datasets for Brain Magnetic Resonance Images

**DOI:** 10.3390/bioengineering11030227

**Published:** 2024-02-27

**Authors:** Seong-Hyeon Kang, Youngjin Lee

**Affiliations:** 1 Department of Biomedical Engineering, Eulji University, Seongnam 13135, Republic of Korea; tjdgus7345@eulji.ac.kr; 2 Department of Radiological Science, Gachon University, Incheon 21936, Republic of Korea

**Keywords:** magnetic resonance imaging, motion artifact, simulation-based dataset, U-Net model

## Abstract

This study aimed to remove motion artifacts from brain magnetic resonance (MR) images using a U-Net model. In addition, a simulation method was proposed to increase the size of the dataset required to train the U-Net model while avoiding the overfitting problem. The volume data were rotated and translated with random intensity and frequency, in three dimensions, and were iterated as the number of slices in the volume data. Then, for every slice, a portion of the motion-free k-space data was replaced with motion k-space data, respectively. In addition, based on the transposed k-space data, we acquired MR images with motion artifacts and residual maps and constructed datasets. For a quantitative evaluation, the root mean square error (RMSE), peak signal-to-noise ratio (PSNR), coefficient of correlation (CC), and universal image quality index (UQI) were measured. The U-Net models for motion artifact reduction with the residual map-based dataset showed the best performance across all evaluation factors. In particular, the RMSE, PSNR, CC, and UQI improved by approximately 5.35×, 1.51×, 1.12×, and 1.01×, respectively, and the U-Net model with the residual map-based dataset was compared with the direct images. In conclusion, our simulation-based dataset demonstrates that U-Net models can be effectively trained for motion artifact reduction.

## 1. Introduction

Magnetic resonance (MR) imaging is used as a noninvasive technique for diagnosing diseases in regions such as brain tissue and blood vessels and the neurological analysis of the human body. In addition, the various sequences and parameters of the MR scanner can be customized for specific purposes, effectively increasing diagnostic accuracy [1,2,3]. However, when acquiring MR images, head movements cause artifacts that can seriously disrupt image analysis [4,5,6,7,8]. In particular, random movements, such as rotation and translation, and periodic movements (i.e., breathing) generate multiple displacements or streak artifacts. This phenomenon is common in patients with diseases that cause involuntary movement, including muscle disorders and neurodegenerative diseases. In addition, movement artifacts are inevitable in patients who lack physical or mental control, particularly children and geriatric patients.

MR images can be acquired using additional guidelines or sedation injections to minimize the generation of motion artifacts. However, repeated imaging increases the overall examination time and inconveniences patients. This can increase the workload because subsequent image processing is not performed. In addition, pediatric and geriatric patients can experience additional side effects, such as toxicity and falls, due to sedation [9,10,11]. To solve the motion artifact problem without sedation, techniques for estimating pulse sequences and motion parameters in real-time, using additional data acquired using sensor-based prospective and retrospective methods, should be considered [12,13,14]. However, this approach incurs an inefficient cost for the additional hardware and requires a large time investment to estimate the variables at play. In addition, raw frequency domain (k-space) data are required for accurate calculations. However, obtaining k-space data when conducting research using public data is difficult. Moreover, although real MR scanners are available, most cannot provide k-space data because of storage and other issues [15,16].

Deep learning-based motion artifact estimation models can solve the problems of conventional sensor-based approaches [17,18,19]. Convolutional neural networks (CNNs) can be efficiently trained using regressive operations by providing before and after images of motion artifacts under identical conditions (i.e., noise intensity, pixel-by-pixel anatomical location, and tissue signal strength). In particular, the U-Net model improves image characteristics and eliminates noise and artifacts in MR images. However, acquiring a large number of datasets from the same environment, using real MR scanners, to train the U-Net model is impractical. In particular, the U-Net model exhibited high performance when provided with ideally paired datasets [20,21,22].

To acquire an idealized dataset that can improve the performance of the U-Net model, various simulation approaches have been proposed. In previous research, simulation-based motion artifact reduction approaches for brain MR images were designed based on rotational and translational motion. In addition, the intensity of the target motion artifact was obtained by controlling the degree of rotation and translational motion. Consequentially, previous research has demonstrated that simulated datasets can be used to train U-Net models that can remove motion artifacts. In addition, these approaches indicate that simulation-based datasets, which estimate the input data based on label data, may be more suitable for training U-Net models than real MR scanner-based datasets. However, many simulation approaches only consider motion in one plane. When acquiring brain MR images, motion along the *z*-axis, such as breathing, swallowing, and nodding, can cause artifacts. Hence, three-dimensional motion should be considered to acquire meaningful datasets that simulate the real clinical environment.

Moreover, training the same U-Net model can lead to different performances due to the computational efficiency of and amount of data required for dataset construction approaches, such as residual or direct image-based methods [23,24,25]. In particular, residual map-based training is more advantageous than direct images in terms of training speed and parameter optimization. In addition, residual map-based training demonstrates a flexibility to adapt its additional mathematical algorithmic methods based on calculation time and technical advantages [26,27,28]. Therefore, a U-Net model is trained using simulation-based datasets of three-dimensional motion artifacts in this study. In addition, we analyzed the variation in the performance of the U-Net model depending on the availability of residual maps.

## 2. Related Works

Motion artifact simulation research is performed to optimize mathematical algorithm parameters, evaluate the feasibility of hardware-based tracking techniques, and construct datasets for deep learning models. Of these objectives, the importance of simulation research for dataset construction is emphasized. These approaches can solve the data acquisition problems in medical imaging, which have various ethical limitations. In addition, the performance of deep learning models, which require paired data (e.g., U-Net, DenseNet, and SegNet), can be significantly improved by constructing an ideal dataset using simulation approaches that can serve that purpose.

Most motion artifact simulations perform rotations and translations of the acquired spatial domain (i.e., MR image) data, followed by a fast Fourier transform (FFT) to distort the frequency domain (i.e., k-space) data. Pawar et al. and Oksuz et al. rotated and translated a two-dimensional MR image within a specified range to obtain the k-space and extract specific lines in the distorted k-space [29,30]. The extracted k-space data were transposed with non-motion k-space data from the same location. The transposed k-space data were the cause of the motion artifacts in the MR images, which were acquired through the inverse FFT. Additionally, Loizillon et al. and Xu et al. similarly worked with MR images in two dimensions, although they transposed part of the data in the original k-space by specifying the ranges of the distorted k-space data rather than extracting lines [31,32]. In addition, stopping or additional movement after the initial movement occurs can be simulated, rather than unnaturally restored after the initial movement occurs, such as in a lines-based approach. However, two-dimensional simulations of motion artifacts are limited because they cannot predict movements along the *z*-axis, such as breathing and jaw movements. To address this problem, Al-Masni et al. and Shaw et al. rotated and translated MR images in all axial directions [33,34]. In addition, the strength of the motion artifact can be controlled by the range it is transposed over the original k-space, including the number of movements, degree of the rotation angle, and pixel shift.

The proposed three-dimensional approach shows motion artifacts that were very similar to the real MR scanner environment. However, previous studies have only performed volume data unit movements due to the purposes and characteristics of their applied models. The U-Net model requires more and varied datasets for better performance. Thus, our motion artifact simulation method generates random volume data motion for each slice of volume data. In particular, when rotational and translational motion occurs in two or more axes, our simulation method can also simulate motion in an oblique direction. This proposed method can increase the volume of a dataset and solve the problem of overfitting with U-Net model due to the uniform movement of volume data. In addition, the parameter values for the intensity of movement were randomized and applied in a relatively wide range to reflect the sudden and large movements of pediatric, geriatric, and athletic patients.

## 3. Materials and Methods

### 3.1. Subsection Brain MR Images Acquisition

We generated simulated motion artifact MR images based on brain MR images ac-quired using a real MR scanner to train a U-Net model in motion artifact reduction. For this purpose, the AD Neuroimaging Initiative (ADNI) database (adni.loni.usc.edu) was used. In this study, T2-weighted brain MR images with a pixel size and slice thickness of 1 mm, matrix size of 256 × 256, and slice number of 68–172 were used. The *z*-axis range of the acquired T2-weighted brain MR images varied for each patient. However, datasets that show anatomically similar structures and ranges should be provided to train the U-Net model efficiently in motion artifact reduction. Based on the center slice that was most widely visible in the lateral ventricle, from an axial view, volume data consisting of 50 slices were extracted. The extraction process was performed on 200 patients, and a total of 10,000 paired data points were constructed. Among these, 7000, 1000, and 2000 paired data points were applied for the training, validation, and testing of the U-Net model for motion artifact reduction, respectively.

### 3.2. Motion Artifact Simulation

**Figure 1:**
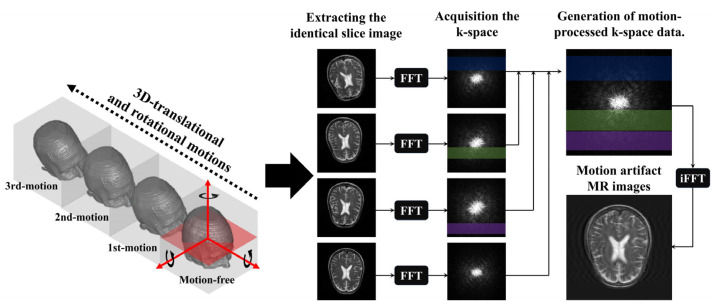
Illustration of the process of simulation-based motion artifact generation in brain magnetic resonance images.

Motion artifacts in MR images are mainly caused by translational and rotational motions, which can be simulated using rotation and pixel-shift methods on the acquired matrix or volume data. Although our proposed U-Net model for motion artifact reduction was trained on a dataset constructed using 2D MR images, the simulation performed using the matrix data extracted from a specific slice of the acquired volume data was considered the rotation (i.e., nodding motion) and translation caused in the *z*-axis. Thus, we transformed the two-dimensional structure of the volume data as shown in Figure 1.

The proposed simulation method randomly rotates and pixel-shifts volume data in the vertical, horizontal, and orthogonal directions. The translation and rotation were per-formed with random values in the range of ±10 pixels and ±5°, respectively. In Figure 1, the red arrows shown on the motion-free image are the three center axes for translation and rotation. The number of movements was between two and four, and subsequent movements were generated via the same simulation process, based on the previous volume data point. Matrix data located on the same *z*-axis were selected from the acquired volume data, and FFTs were performed to create the k-space data. Subsequently, a portion of the motion-free k-space data was replaced with distorted k-space data. To transform the k-space data, motion was assumed to occur at each TR based on a fast spin–echo sequence. This implies that the transformation of the k-space data was performed in the phase-encoded direction. However, motion artifacts could create unrecoverable or unpredictable data in the MR images when the k-space data were improperly transposed. In particular, data located in the center of k-space have an enormous impact on the contrast and anatomical information in MR images. Because of these features, when most of the data located in the center of the motion-free k-space were replaced by distorted k-space data, the U-Net model confused the post-motion anatomy with its pre-motion position. Hence, we assumed that the first movement occurred after more than half of the motion-free k-space data were acquired to generate motion artifacts that can be applied to the dataset. In addition, the TRs in which the movements occurred were randomly selected and not restored to their previous or initial positions after a specific motion occurred. Finally, an inverse FFT was applied to the transformed k-space data to acquire MR images with motion artifacts that could be used as input data. Residual maps of motion-free and motion artifact MR images were obtained to construct additional input data.

### 3.3. U-Net Model for Motion Artifact Reduction

**Figure 2:**
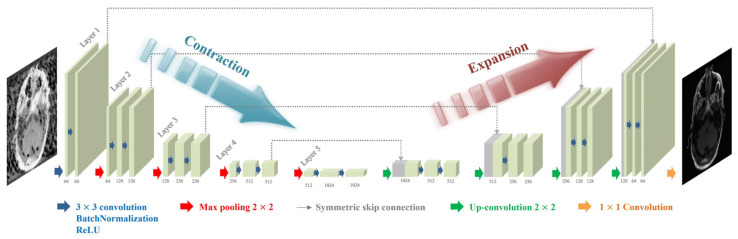
Illustration of the U-Net model for motion artifact reduction.

Figure 2 illustrates the proposed U-Net model for reducing motion artifacts. The structure of the U-Net model consists of a contraction path that extracts and compresses the features that are essential for removing motion artifacts and an expansion path that provides location information. In addition, the parameters for the U-Net models were determined empirically based on a specific previous study [35]. A 3 × 3 convolution was performed twice for each layer of the contraction path, followed by a rectified linear unit (ReLU) and batch normalization (BN). Subsequently, max pooling with a stride value of two sets was performed for downsampling. After acquiring 64 feature maps in the first layer, the number of feature maps was doubled in the next step. For each layer in the expansion path, a 3 × 3 convolution was performed twice, similar to the contraction path, followed by the ReLU and BN. Subsequently, upsampling with a stride value of 2 was applied. As the operation for each layer was performed, the number of feature maps was reduced by half to obtain 64. Finally, a 1 × 1 convolution layer was scaled up to derive the output image. In addition, a skip connection was applied to compensate for the information lost through each layer and allow for faster training. The L2-norm and Adam (adaptive moment estimation) optimizer loss functions were applied to train the U-Net model to reduce motion artifacts. The learning rate and number of epochs were set to 0.0005 and 300, respectively.

### 3.4. Quantitative Evaluation

To evaluate the performance of the U-Net model for motion artifact reduction, we performed a similarity evaluation between the output and label images without motion artifacts. For these evaluations, the root mean square error (RMSE), peak signal-to-noise ratio (PSNR), coefficient of correlation (CC), and universal image quality index (UQI) were measured as follows:

$$RMSE=\sqrt{\frac{\sum_{i=1}^{N}({f_{i}-g_{i})}^{2}}{N}}$$

$$PSNR=10{log}_{10}(\frac{{S_{peak}}^{2}}{{RMSE}^{2}})$$

where $f_{i}$ and $g_{i}$ represent the reference and comparison images, respectively; N is the number of pixels in the image; and ${S_{peak}}^{2}$ is the maximum signal intensity in the region of interest.

$$CC=\frac{\sum_{i=1}^{N}\left(f_{i}-\hat{f})(g_{i}-\hat{g}\right)}{\sqrt{\sum_{i=1}^{N}{\left(f_{i}-\hat{f}\right)}^{2}}\sqrt{\sum_{i=1}^{N}{\left(g_{i}-\hat{g}\right)}^{2}}}$$

$$UQI=\frac{4\mu_{f}\mu_{g}\sigma_{fg}}{\left({\mu_{f}}^{2}+{\mu_{g}}^{2})({\sigma_{f}}^{2}+{\sigma_{g}}^{2}\right)}$$

where $\hat{f}$ and $\hat{g}$ represent the average pixel values of the reference and comparison images, respectively; $\mu_{f}$ and $\mu_{g}$ represent the average luminance values, respectively; and $\sigma_{fg}$ represents the covariance between the two images.

## 4. Results

**Figure 3:**
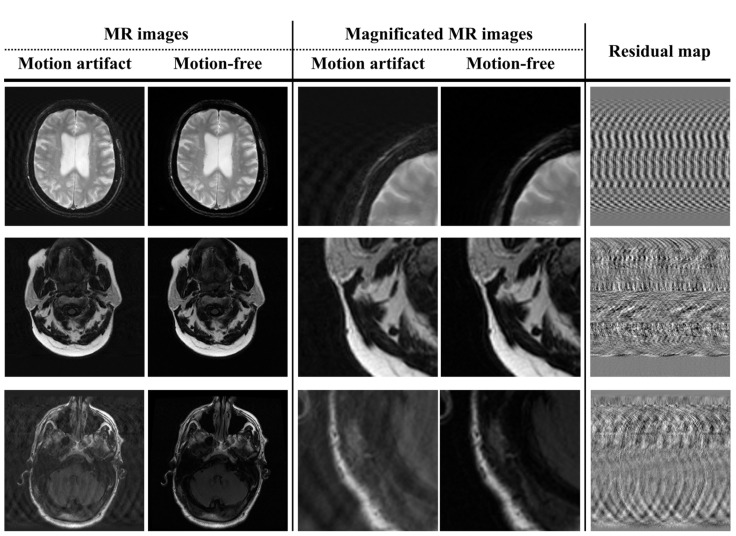
Simulation-based motion artifacts and motion-free magnetic resonance images with residual maps.

Figure 3 shows the simulated motion artifact images (i.e., direct images) and residual maps from the motion-free brain MR images. Motion artifacts were confirmed to be generated in the direction of the phase encoding. Furthermore, the motion artifact patterns observed in the residual maps show that the proposed method can simulate the intensities of different motion artifacts. Based on the acquired direct images and residual maps, two datasets were constructed and used to train the U-Net model in motion artifact reduction.

**Figure 4:**
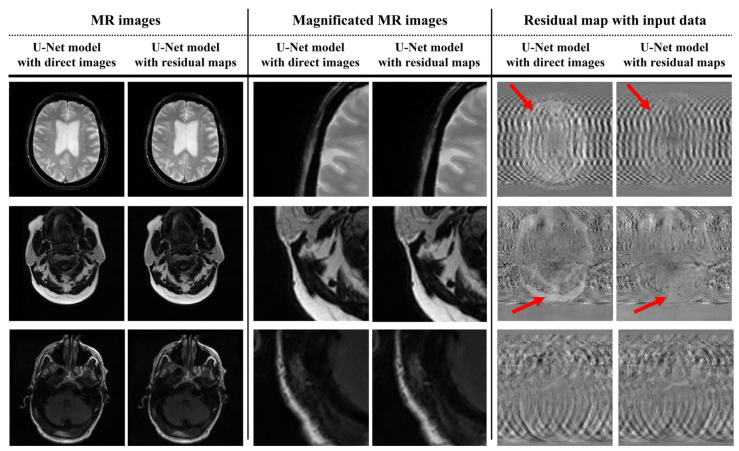
Results of applying a U-Net model for motion artifact reduction of MR images with simulation-based motion artifacts.

**Figure 5:**
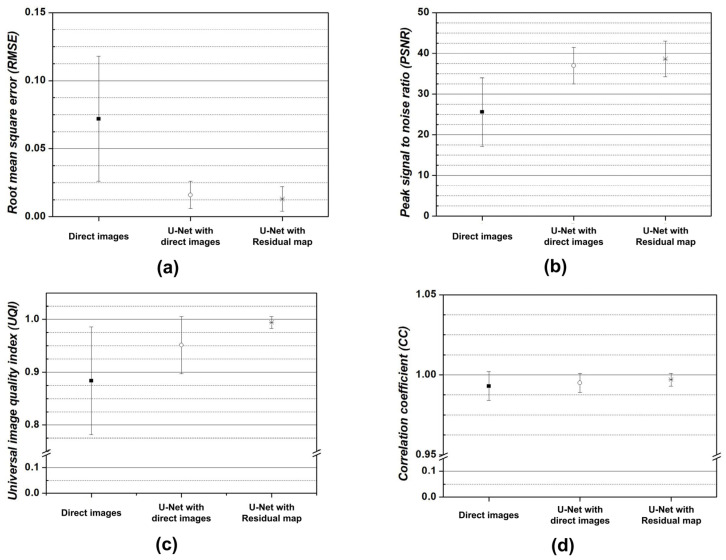
Results of quantitative evaluation for reconstructed MR images with U-Net models for motion artifact reduction: (**a**) Root mean square error (RMSE), (**b**) peak signal-to-noise ratio (PSNR), (**c**) universal image quality index (UQI), and (**d**) correlation coefficient (CC).

Figure 4 shows the results of applying the U-Net model for motion artifact reduction to each trained dataset. In addition, the U-Net models overcome the decreased contrast caused by the distortion of the direct current and the low-frequency signals in the k-space data. Additionally, noise reduction was confirmed in the subject and background regions. However, compared to motion-free images, motion artifacts were not completely removed from certain regions, such as the edges of the tissues and near the eyeballs. These phenomena were confirmed visually using a residual map. We evaluated image similarity to analyze the performance of the U-Net model in motion artifact reduction. Figure 5 shows the quantitative evaluation results of the U-Net model for motion artifact reduction using datasets based on direct images and residual maps that include direct images.

Consequently, the RMSE, PSNR, UQI, and CC for the direct images generated through motion artifact simulation were approximately 0.072 ± 0.046, 25.56 ± 8.40, 0.884 ± 0.102, and 0.993 ± 0.009, respectively. In addition, the results were improved compared with direct images for all evaluation factors when applying the U-Net model for motion artifact reduction, regardless of the dataset type. In particular, the U-Net model for motion artifact reduction, on datasets based on residual maps and direct images, improved the RMSE by approximately 5.35× and 4.42× and the PSNR by approximately 1.51× and 1.44×, respectively, compared to the dataset of direct images. In addition, the UQI and CC of the U-Net model for motion artifact reduction in terms of the residual map-based dataset were approximately 0.994 ± 0.011 and 0.997 ± 0.004, respectively, compared to the direct image-based dataset, for which they were approximately 0.951 ± 0.054 and 0.995 ± 0.006, respectively. In addition, paired *t*-tests were performed for direct images, the U-Net model with direct images, and the U-Net model with residual maps, respectively, and the *p* values were less than 0.01 for all evaluation factors.

## 5. Discussion

The purpose of this study is to propose a U-Net model that can solve the problem of motion artifacts in brain MR images. For the efficient training of the U-Net model, paired data points with precisely the same anatomy, except for motion artifacts, were required. To overcome these limitations, we conducted a simulation process to predict the rotational and translational motion of three-dimensional volume data. In addition, various random variables were applied to generate datasets with varying intensities of motion artifacts to prevent performance overestimation due to overfitting the U-Net model. Moreover, each dataset, which consisted of direct images and residual maps, was constructed and applied to analyze the performance change in the U-Net model for motion artifact reduction according to the type of dataset used [36,37,38,39]. In this study, the intensity and number of movements were limited by the hardware performance and by randomizing the variables that controlled data generation. However, the results of our motion artifact simulation, presented in Figure 3, demonstrate the feasibility of obtaining large amounts of data by densely setting various parameters within a certain range. In particular, these processes should be considered for deep learning models that can be trained based on 3D volume data because of the inclusion of *z*-axis motion.

Figure 3 and Figure 4 provide a visual analysis of the ability of the U-Net models to compare the motion-free and motion artifact images. The output images derived using the U-Net models exhibited an improved performance in terms of motion artifact reduction. In particular, in this process, we applied U-Net models and confirmed that this process was accompanied by noise reduction. Since noise reduction can improve the signal-to-noise ratio of MR images, the application of U-Net models shows additional positive potential, including the use of inexpensive low-magnetic-field MR scanners and short acquisition times for images. In addition, these features provide us with the optional flexibility to apply additional image processing techniques which require a worsening of the SNR, such as super resolution and deblurring [40,41,42,43]. However, smoothing implies that some data from a group might be distorted or removed. These concerns are reinforced in brain MR images with thin or small bones and tissues. Thus, retrospective research using MR images with better characteristics, such as a 3.0T MR scanner, should be performed to analyze this accurately [44,45,46].

In addition, image similarity evaluations were performed with 2000 paired data points, and mean and standard deviation values were presented. Figure 5 also shows that a dataset based on residual maps can improve the performance of a U-Net model more than direct images. Direct images contain variable information such as the tissue signal, contrast, noise, and motion artifacts. Thus, a direct image-based U-Net model shows inefficient performance with the same depth of layers because it considers various features to estimate the output image. In contrast, residual maps only contain information about motion artifacts, although a small amount of random tissue and noise signals can remain. This means that the performance of a residual map-based U-Net model can improve efficiently because only a limited specificity is estimated [47,48].

The residual maps in Figure 3 show that the simulated motion artifact repeats a specific signal in the phase encoding direction. In addition, the residual maps in Figure 4 show the results estimated by the U-Net model in terms of motion artifact reduction. However, the direct image-based U-Net model shows that its performance in detecting repetitive signals deteriorates in the subject region compared to the residual-ap-based U-net model. Specifically, the region marked by the red arrow in Figure 4 shows an excessive decrease in signal intensity. This phenomenon prevents the analysis of motion artifact patterns and means that the signal changes of subjects caused by a distortion in the low-frequency region of the k-space cannot be accurately reconstructed. Furthermore, the performance difference between the two U-Net models increases when motion artifact reduction is performed on T2-weighted images due to the cerebrospinal fluid and eyeballs, which are described as high-intensity signals. In contrast, the performance differences between the U-Net models are difficult to analyze clearly in brain MR images which have a low proportion of high-intensity signals, as shown in the last image in Figure 4. Thus, the performance of the residual map-based U-Net model might not significantly improve, compared to the direct image-based U-Net model, in T1-weighted brain MR images with less tissue and a high-intensity signal, such as bone. In addition, the standard deviation and mean values of the quantitative evaluation factors of the residual map-based U-Net model were improved compared to those of the direct image-based U-Net model (Figure 5). These results show that the residual map-based U-Net model has better performance. In particular, the low standard deviation values of the quantitative evaluation factors show that there is a possibility a greater improvement of the reproducibility and stability of the residual map-based U-Net model, in MR images with few high-intensity signals.

Recently, various methods using deep learning-based models have been proposed to overcome the limitations of conventional methods in the reduction of motion artifacts in MR images [49]. Of the conventional methods, sensor-based tracking techniques have been effectively applied to prevent the motion artifacts caused by breathing during abdominal and chest MR imaging [50,51]. However, this technique requires additional hardware and cannot achieve its expected performance for irregular movements. In addition, low-quality MR images were obtained because data can only be acquired for a limited period of time. Additionally, its applicable environment is limited, such as to eyeball movement, which causes motion artifacts in brain MR images [52]. To solve these problems, accelerated k-space data acquisition and image processing techniques based on mathematical reconstruction algorithms, such as compressed sensing, should be considered [53,54]. However, reconstruction algorithm-based techniques are difficult to optimize due to variable parameters and the MR acquisition environment. In addition, a real MR scanner capable of providing k-space data and with a large amount of storage should be available, since k-space data were required for these mathematical computations. In contrast, deep learning-based models have been actively researched due to their superior performance and potential [55]. Among these models, generative adversarial network (GAN)-based models can effectively remove motion artifacts by combining a generator and a discriminator [56,57]. In particular, GAN models show improved performance compared to other models when trained using limited data. In addition, they are highly convenient, due to their unsupervised learning and application of various types of data. However, when a large amount of data can be provided, the U-Net-based model for medical image processing shows superior performance [5,8,21]. In particular, simulation-based studies, which can construct large datasets with various variables, prefer the U-Net model compared to other models.

Overall, the simulated motion artifact-based dataset shows that it was effective for training U-Net models [58,59,60,61]. In particular, the residual map-based U-Net model shows high performance and reproducibility in motion artifact reduction. However, an inaccurate motion artifact reduction performance in the subject region, unintended smoothing, and signal loss of tissues must be resolved for its effective clinical application. In addition, our simulation method was able to obtain MR images with motion artifacts from various rotational and translational movements. However, although the intensity and number of movements were changed, it is difficult to continuously generate large changes from the initial MR images. These results mean that, although our simulation method can generate large amounts of data to train the U-Net model, we should be concerned about the overestimating of its performance due to overfitting [62,63]. To analyze the impact of potential problems with the proposed simulation method, a comparative evaluation with other motion artifact techniques and simulation methods should be performed. However, since this study only presents comparative results for dataset construction methods, some results of our proposed simulation method may have limitations.

**Figure 6:**
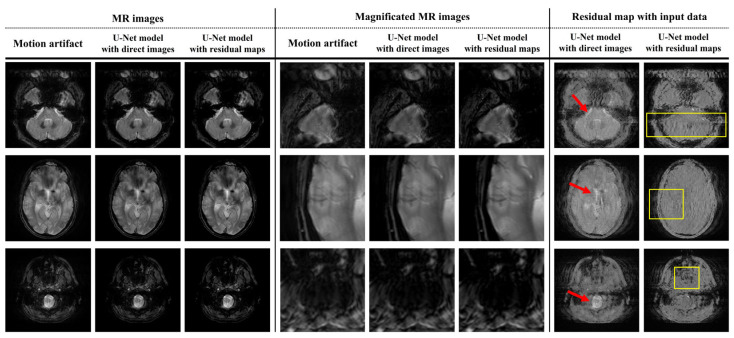
Results of applying U-Net models to real MR images with motion artifacts from ADNI database.

In addition, the proposed simulation method significantly increases the processing time compared to the conventional simulation method, because the computation based on volume data should be performed repeatedly. Moreover, further research, with large data-driven training and testing, K-fold cross-validation, etc., should be considered to accurately analyze the performance of the proposed model. In addition, the proposed U-Net models were only trained on a simulation-based dataset using ADNI data. Hence, additional analysis was required to evaluate the feasibility of the proposed U-Net models in terms of real MR images. For this purpose, we collected the MR images, with motion artifacts, of five patients (100 slices) from the ADNI database. Figure 6 shows the results of applying the U-Net models to real MR images with motion artifacts. Visually, the residual map-based U-Net model shows a performance improvement compared to the direct image-based U-Net model. In particular, the red arrows in Figure 6 show the excessive loss of high-intensity signals when applying the direct image-based U-Net model identically to how it was applied the simulation experiments. In addition, the yellow boxes in Figure 6 show that regular patterns (i.e., motion artifacts) were more efficiently extracted by the residual map-based U-Net model than direct images.

**Figure 7:**
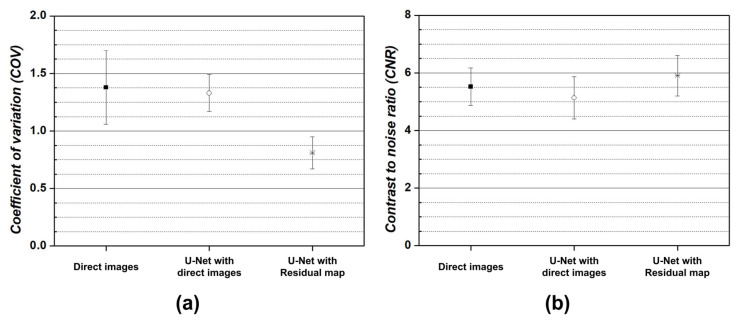
Results of the (**a**) coefficient of variation (COV) and (**b**) contrast to noise ratio (CNR) of real MR images with motion artifacts, to which U-Net models were applied.

Quantitative analysis using image similarity factors was impossible because the ground-truth could not be estimated for the real MR images with motion artifacts. However, multiple displacements or streak artifacts caused by movement randomly change the signal intensity of homogeneous tissue and the background (i.e., air). Considering these features, the coefficient of variation (COV) and the contrast to noise ratio (CNR) were measured to indirectly evaluate the performance of the U-Net models using segmented background and gray matter signals via k-means clustering, as follows:

$$COV=\frac{\sigma_{b}}{\mu_{b}}$$

$$CNR=\frac{\mu_{t}-\mu_{b}}{\sqrt{\sigma_{t}^{2}+\sigma_{b}^{2}}}$$

where $\sigma_{b}$ and $\mu_{b}$ are the mean and standard deviation of the background signal intensities and $\sigma_{t}$ and $\mu_{t}$ are the mean and standard deviation of gray matter. Figure 7 shows the COV and CNR results of the performance evaluation of the U-Net model on real MR images with motion artifacts. A lower COV value for the background indicated the more effective U-Net model for motion artifact reduction. As a result, the direct image- and residual map-based U-Net models show that the motion artifacts were removed from the real MR images. In particular, the COV of the residual map-based U-Net model shows an improvement of approximately 1.64× compared to the direct image. In addition, the CNR was measured to analyze the composite performance of the U-Net models, which considers the degree of signal maintenance and motion artifact reduction in the tissue. The residual map-based U-Net model shows an approximately 1.07× improvement compared to the real MR image. However, in the direct image-based U-Net model, the lowest CNR values were measured. Although the U-Net model solved the motion artifact problem, it was discovered that the image contrast was degraded due to excessive signal loss.

## 6. Conclusions

In this study, we applied U-Net models to reduce the motion artifacts in brain MR images. In addition, we generated motion artifacts based on simulations to construct an ideal paired-data-based dataset. In conclusion, the simulation-based dataset effectively trained the U-Net models to reduce motion artifacts. Additionally, datasets with appropriate preprocessing could further enhance the performance of the U-Net models.

## Data Availability

The data used in the preparation of this article were obtained from the ADNI database (adni.loni.usc.edu, accessed on 31 October 2023). The ADNI was launched in 2003 as a public–private partnership led by Principal Investigator Michael W. Weiner. The primary goal of the ADNI has been to test whether serial magnetic resonance imaging (MRI), positron emission tomography (PET), other biological markers, and clinical and neuropsychological assessments can be combined to measure the progression of mild cognitive impairment (MCI) and early Alzheimer’s disease (AD). For up-to-date information, see http://adni-info.org/ (accessed on 31 October 2023). The data used in the preparation of this article were obtained from the Alzheimer’s Disease Neuroimaging Initiative (ADNI) database (https://adni.loni.usc.edu, accessed on 31 October 2023). As such, the investigators within the ADNI contributed to the design and implementation of the ADNI and/or provided data but did not participate in the analysis or writing of this report. A complete listing of the ADNI investigators can be found at https://adni.loni.usc.edu/wpcontent/uploads/how to apply/ADNI Acknowledgement List.pdf (accessed on 31 October 2023).
